# BgCut: Automatic Ship Detection from UAV Images

**DOI:** 10.1155/2014/171978

**Published:** 2014-04-03

**Authors:** Chao Xu, Dongping Zhang, Zhengning Zhang, Zhiyong Feng

**Affiliations:** ^1^School of Computer Software, Tianjin University, Tianjin 300072, China; ^2^Space Star Technology Co., Ltd., Beijing 100086, China

## Abstract

Ship detection in static UAV aerial images is a fundamental challenge in sea target detection and precise positioning. In this paper, an improved universal background model based on Grabcut algorithm is proposed to segment foreground objects from sea automatically. First, a sea template library including images in different natural conditions is built to provide an initial template to the model. Then the background trimap is obtained by combing some templates matching with region growing algorithm. The output trimap initializes Grabcut background instead of manual intervention and the process of segmentation without iteration. The effectiveness of our proposed model is demonstrated by extensive experiments on a certain area of real UAV aerial images by an airborne Canon 5D Mark. The proposed algorithm is not only adaptive but also with good segmentation. Furthermore, the model in this paper can be well applied in the automated processing of industrial images for related researches.

## 1. Introduction

With the application of unmanned aerial vehicles (UAV) in the supervision of ships, forestry, and natural resources [1], the use of UAV in positioning of vessels and management of fishery activities is confirmed [2]. UAV, according to the target task, can carry different devices such as synthetic aperture radar (SAR) systems and high-resolution optical camera aerial system. Compared with SAR images, UAV aerial images have several advantages, including high resolution and definition, and measurable character [3]. Due to the large amount of big-data images acquired during one flight, classical manual or semisupervised image processing method is no longer suitable or applicable for some specific conditions and surroundings. Therefore, automatic object identification method is crucial to industrial images processing. Since automated processing often loses recognition accuracy, object recognition and localization have become a hot issue in aerial images on the premise of ensuring high accuracy. For these reasons, we consider that the problem can also be regarded as separation of sea foreground and background. Because the background of the sea varies greatly with the natural conditions such as illumination, weather, and wind speed, building a generic background model for high-accuracy automatic target recognition is of great significance.

Considering the problems mentioned above, we design and propose an improved universal background model based on Grabcut algorithm to separate and identify ship candidate region automatically and quickly. Meanwhile, pyramid multiresolving can be applied for the lowest level images as the advanced processing. Not only can the background model guarantee the recognition rate for surface extraction, but also it covers the shortage that Grabcut algorithm in the image segmentation process cannot automatically obtain the background results.

This paper is arranged as follows. Section 2 is a brief introduction to ship identification methods and development of the Grabcut algorithm.  Section 3 is the details of our proposed algorithm. Discussion and analysis of experiments and results are shown in Section 4.

## 2. Related Work

At present, the recognition for ship identification in the UAV aerial images is still an important field; yet it is a hot issue for research on SAR images and inverse synthetic aperture radar (ISAR) images to identity the ship target.

Ship identification methods are mainly divided into three parts, which are the coarse-segmentation-based feature extraction, the statistic-model-based constant false alarm rate (CFAR) algorithm, and the direct image segmentation method. First of all, the coarse-segmentation-based feature extraction methods include single feature and multifeatures. The single feature is common, such as building a composite distribution to simulate the characteristic of the various types of sea surface [4] and applying texture descriptors to extract feature to build statistical matrix [5]. The multifeatures method is different, for example, some multifeatures can be obtained from the combination of the aspect ratio and the number of target pixels [6]. These kinds of recognition methods are limited by the feature library completeness and features selection and some even rely on a priori parameters or previous segmentation.

CFAR based on the statistical model is the most widely used algorithm in SAR image target detection, and some fast algorithms based on it increase the unsatisfactory calculation speed [7], such as the Gama-CFAR [8] and weighted Parzen window clustering algorithm [9]. Even though fast algorithms improve the processing speed, its prescreening results are not rejoined, and it cannot ensure high detection accuracy [7].

The direct image segmentation method consists of traditional approaches and some combine specific theories and models. Traditional ones are mainly region based and edge based; model-based methods are such as triplet Markov fields [10], the layer set method [11], and snake model [12]; theory-based methods are such as fuzzy c-means clustering algorithm [13] and fuzzy logic [14]; graph-based methods are such as graph cut [15, 16], Grabcut [17], and normalized cut [18, 19]. Nevertheless, model-based methodology is usually suitable for single-object segmentation; edge-based approach is not strong in noise adaptability; region-based mechanism relies on interaction or a priori conditions. Method based on graph theory requires human interaction; however, its high accuracy meets the processing demands of the aerial image perfectly.

Because of its major advantage, graph cut algorithm is widely used in kinds of fields, such as the myocardium image segmentation [20], accurate segmentation of mural images [21], buildings in optical satellite images [22], and the SAR image change detection [23]. SAR image target segmentation [24] uses combination of Grabcut algorithm and neighborhood growing algorithm. While ships in UAV aerial images have different sizes and variable direction characteristics, most of the methods may omit some small fishing boats as spot which affect the whole and final recognition rate.

Currently Grabcut-based image segmentation methods mainly make improvements in energy minimization function and minimizing the objective function. Global energy minimization methods include simulated annealing, dynamic programming, and graph theory; local energy minimization methods include variation and ICM methods [25]. Sometimes, it is essential to construct a good initial model for this sensitive processing. For example, it is impossible to offer an optimal trimap manually [26]. In order to solve the problems of the initialization, the adaptive shape prior method provides a priori trimap to Graphcut [27], which is particular for specific shapes so that this kind of model lacks the generality and ability to detect various shapes of vessels.

In response to this situation, we propose a universal background model to initialize and optimize the Grabcut algorithm. The improved algorithm is adaptable and suitable to kinds of sea surfaces, which is robust to different shapes and sizes of ships. The novelty of our approach lies first in this universal background model. We apply a sea template library to enhance the generality by randomly selecting pixel points of the sea as the feature image and automatically acquiring the new generated template. Secondly, template matching algorithm is designed to find a certain sea seed point which can grow out optimal background area and generate background trimap. Finally, we use the trimap to optimize and improve Grabcut and to segment the foreground objects.

## 3. Universal Background Model with Grabcut

In the present paper, the proposed algorithm is divided into two main sections: building a background model and improving Grabcut segmentation. Background model consists of three parts. Firstly, we use the sum of squared difference (SSD) [28] template matching based on selected sea template to acquire seed point. Secondly, we apply the neighborhood growing algorithm based on the seed point to carry out the growth of the sea surface background and to get background mask image. Finally, we transform the mask image and generate background trimap images. Since trimap initializes the Grabcut background model, there is one process that can obtain higher accurate results.

### 3.1. Automatic Selection of Sea Template

#### 3.1.1. Template Library Building

**Figure 1:**
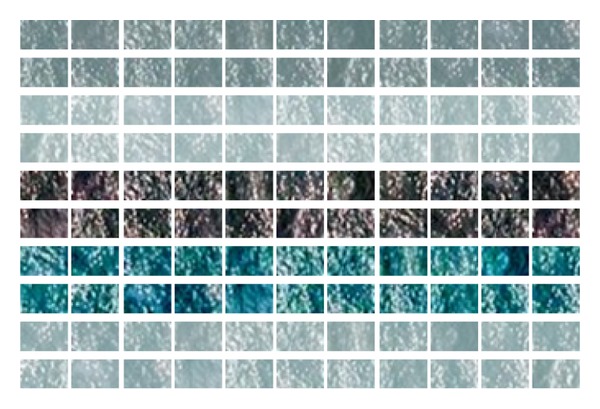
Part of sea template library. The first two rows represent the cloudy condition, the next two rows represent the foggy condition, the fifth and the sixth rows represent the night condition, the seventh and the eighth rows represent the rainy condition, and the last two rows represent the sunny condition.

The sea template library includes samples from different sea areas, season, weather, and natural conditions. The size of samples is 26∗16 and the number is 1000. Partial template samples are shown in Figure 1, including collection in cloudy day, foggy day, rainy day, sunny day, and night.

#### 3.1.2. Automatic Template Selection

We assume that the number of background pixels is much larger than the number of foreground pixels. The parameter *m* is the number of feature samples. *m* blocks of pixels are randomly selected from the image to be segmented with size of 10∗5. Each pixel block is called a feature image sample. Respectively, the characteristics of the image can be calculated as the mean gray value $\bar{G}(p)$, the mean brightness $\bar{V}(p)$, and the mean hue $\bar{H}(p)$. Equation (1) is to calculate the whole characteristic value of the image as follows:

(1) $$\begin{array}{c} M\left(p\right)=\alpha \bar{G}\left(p\right)+\beta \bar{V}\left(p\right)+\left(1-\alpha -\beta \right)\bar{H}\left(p\right), \end{array}$$

where *M*(*p*) is the characteristic value of the feature sample. *α* and *β* are the positive weights of *α*, *β* ∈ (0,1) and *α* + *β* < 1. After that the obtained characteristic value can be calculated and matched with all of the templates, which is defined as

(2) $$\begin{array}{c} \delta_{ij}=||M_{i}\left(p_{i}\right)-M_{j}\left(q_{j}\right)||, \end{array}$$

where *i* ∈ (1, *m*)  and *j* ∈ (1, *n*). We assign *m* = 20 and *n* = 1000 in this paper. The feature sample set *P* = (*p*_1_,…, *p*_*i*_,…, *p*_*m*_) has *m* samples, and *p*_*i*_ is the *i*th feature sample. The template library *Q* = (*q*_1_,…, *q*_*j*_,…, *q*_*n*_) has *n* samples, and *q*_*j*_ is the *j*th template. Evidently, *δ*_*ij*_ represents the degree of differences between the feature sample and some template samples.

Then, a function *C*(*δ*_*ij*_) is defined to sum up the number of different *δ*_*ij*_, and the voting method shown in (3) is adopted to find out the most similar template as *j*

(3) $$\begin{array}{c} j:=\arg\;\underset{\begin{array}{c} i=1,m\\ j=1,n \end{array}}{\max}C\left(\delta_{ij}\right). \end{array}$$

The task is to make sure that a suitable template will be selected to seek seed point and to initialize the background model.

### 3.2. Automatic Trimap by Background Model

According to the analysis mentioned above, trimap is specified as *T* = {*T*_*B*_, *T*_*F*_, *T*_*U*_} in which background regions *T*_*B*_ and foreground regions *T*_*F*_ are marked by Grabcut algorithm [17]. To get more reasonable results, unknown regions *T*_*U*_ in trimap should be as little as possible. The reason is that color and mark information of the adjacent pixels is used to estimate and to identify whether the unknown region pixels are background or foreground. If there are too many unknown regions or the regions are too far away from the marked area, it will greatly reduce the accuracy of sampling-based assessment and even get error results. Intuitively, some regions are not divided or processed.

This section describes the parts of trimap modeling automatically, which is mainly divided into three steps: finding a better seed point by SSD template matching algorithm, growing an optimal background mask with the seed point, and generating background trimap.

#### 3.2.1. SSD Template Matching Algorithm

Sum of squared difference (SSD) [28] is one of template matching methods defined as (4). As we know that the best matching value is 0, because the larger the value is, the worse the match will be. Consider

(4) $$\begin{array}{c} \text{SSD}\left(x,y\right)=\frac{\sum_{x^{\prime },y^{\prime }}^{}{\left[Q\left(x^{\prime },y^{\prime }\right)-I\left(x+x^{\prime },y+y^{\prime }\right)\right]}^{2}}{\sqrt{\sum_{x^{\prime }y^{\prime }}^{}Q{\left(x^{\prime },y^{\prime }\right)}^{2}\sum_{x^{\prime }y^{\prime }}^{}I{\left(x+x^{\prime },y+y^{\prime }\right)}^{2}}}, \end{array}$$

where *Q* is the sea template image and *I* is the input target image. *x*′ ∈ (0, *w* − 1) and *y*′ ∈ (0, *h* − 1); *w* is the width of *Q* and *h* is the height of *Q*. According to (4), the position point (*x*, *y*) with the minimum SSD can be selected as the seed value.

#### 3.2.2. Optimal Background Growth

The obtained seed point from the previous section is passed to the neighborhood growing algorithm as the growth starting point. And the optimal background should be identified for more than 90 of the total image background. The region growing algorithm process is described in Algorithm 1.

In the optimal algorithm, the prior threshold value is set to be 0.07, which is obtained by a large number of tests on the sea images. Obviously, the threshold value depends on the resolution of the target images.

#### 3.2.3. Trimap via Background Mask

The background region obtained from the growth of the growing algorithm scatters to be punctate; however, it is not the target trimap image. This mask has three channels with RGB color. We can map it to a single channel of gray-scale image which is the result trimap. The mask image is *L* = (*l*_1_,…, *l*_*n*_,…, *l*_*N*_), and the map function is shown as

(5) $$\begin{array}{c} T_{n}=\{\begin{array}{cc} 0 & l_{n}\left(1\right)=0,\\ 3 & l_{n}\left(1\right)\neq 0, \end{array} \end{array}$$

where *l*_*n*_(*i*) presents the value of the *i*th dimension of the pixel *l*_*n*_ and *T*_*n*_ is the pixel of trimap.

Compared to the trimap described as *T* = {*T*_*B*_, *T*_*F*_, *T*_*U*_} [17], we redefine the trimap as *T* = {*T*_*B*_, *T*_PF_}, where *T*_*B*_ still represents the background and *T*_PF_ represents the possible foreground. In this paper, we assume that *T*_PF_ = 3, and it is a further refinement calibration to *T*_*U*_, which means a greater possibility of the pixel belonging to the foreground. The initial value of *T*_*B*_ and *T*_*F*_ in trimap is set to be fixable, while *T*_*U*_ is allocated by the Gaussian mixture models (GMM). There are two types of connections in GMM model including *N*-Links and *T*-Links [29]. *N*-Links connect the neighboring pixels, which describes the cost of division boundary between adjacent pixels. *T*-Links connect the nodes of the background and foreground, which describes the possibility of unknown pixel belonging to the foreground or background. As we know, T-link weights can be upgraded by GMM [29]. Therefore, *T*_PF_ is applied, and its initial value will be reallocated after the GMM model recalculation.

This model can obtain the sea background mask automatically, and the mask image is the optimal result. For some reason, the optimal model should combine Grabcut algorithm in the further processing step, if high-precision region segmentation is wanted. Grabcut will make a scattered background mask connection and even achieve high precise extraction for foreground target area.

### 3.3. Grabcut Algorithm Based on Background Model

In order to achieve automatic target foreground segmentation, the key issue is how to design the combination of the improved algorithm of Grabcut background model. From the view of characteristics of the original Grabcut algorithm, it can achieve high precise segmentation of foreground from background; however, it needs human intervention and interaction. Considering the industrial image processing, it is necessary to improve the original algorithm by initializing background model automatically. Therefore, the new and improved processing flow of Grabcut is shown in Algorithm of BgCut.

*Algorithm *of BgCut-improved Grabcut algorithm*. Input*: Trimap, *T*_*B*_ = 0, *T*_PF_ = 3.

(i) Establish the initial segmentation from Trimap. Assign pixels in *T*_*B*_ to TrimBackground which is the set of background points; assign pixels in *T*_PF_ to TrimForeground which is the set of foreground points.
(ii) Initialize *α*_*m*_ = 0 for *m* ∈ *T*_*B*_ and *α*_*m*_ = 3 for *m* ∈ *T*_PF_, where *α*_*m*_ is the opacity value of the pixel *m*, which is used to donate the initial trimap *T*.
(iii) Use the initial segmentation to obtain GMMs parameters.
 
*N*-Links between pixel *m* and pixel *n* are shown as follows:
  (6) $$\begin{array}{c} N\left(m,n\right)=\frac{\text{Aplha}}{\mathrm{dist}\left(m,n\right)}e^{-\beta {||z_{m}-z_{n}||}^{2}}, \end{array}$$
 where Alpha is a prior parameter, and its value is usually set to be 50. dist⁡() is the Euclidean distance between pixel *m* and pixel *n*, and *z*_*m*_ and *z*_*n*_ are the color values [29].
 
*T*-Links for pixel *m*, the similar to *N*-Links, are shown as follows:
  (7) D(m)=−log⁡[π(αm,i)∗e((1/2)[zm−μ(αm,i)]T∑(αm,i)−1)[zm−μ(αm,i)]det⁡∑(αm,i)],
 where *π*() is the mixture weighting coefficient and mean *μ* and covariance Σ are of the GMM components for the background and foreground distribution. It is a restatement of *T*-Links in reference [17]. Consider
  (8) $$\begin{array}{c} \theta :=\arg\;\min U\left(\alpha ,k,\theta ,z\right), \end{array}$$
 where *k* ∈ {1,…, *K*} is a vector and *K* is the number of GMM background and foreground components. *θ* is the parameter of GMM which is learned from data *z*.
(iv) Build new GMMs after the iteration and calculation. Calculate the minimum value of Gibbs energy function: *E*(*α*, *k*, *θ*, *z*) and then obtain the min cut:
  (9) $$\begin{array}{c} E\left(m,n\right)=U\left(m\right)+N\left(m,n\right), \end{array}$$
 where *U*(*m*) = ∑_*k*_ *D*(*m*), which is data items of the Color GMM and depends on the GMM components *k*.
(v) Apply border matting.

Improved Grabcut algorithm based on background model can achieve background trimap automatically, and it will meet the demand for automatic segmentation of big-data image. In this paper, the Gibbs is applied as energy function, and the application of the maximized flow can be used to minimum the cut method, which guarantees the accuracy of the segmentation. Above all, our proposed model upgrades the initialization process and the quality of background trimap, and it will achieve more accurate identification results for sea target.

## 4. Experiment

Experiments are conducted to evaluate the proposed algorithm with the real UAV aerial images for the purpose of automatic foreground target segmentation. All the experimental images are collected and obtained from a certain area of the East China Sea in 2012. Canon 5D Mark is used as the camera on the UAV with the flying height of 800 m, and the resolution of the image is 0.1 m. All the images have been preprocessed such as defogging and uniforming light. Then the big-data images are stored in kmz format which are compressed by pyramid hierarchy mechanism. Experiment is performed on the 8th layer, which is the bottom layer of images after decompression so as to verify the improved BgCut model in this paper.

The condition of modeling and calculation is CPU 2 GHz and memory is 2 GB. And the seed point can be calculated by the OpenCV library, which can be downloaded from “http://sourceforge.net/projects/opencvlibrary/files/.”

### 4.1. Results and Conclusions

For our proposed algorithm BgCut for the experiment, it is a graphical improved modeling and segmenting approach which can realize the whole task automatically. Experimental process can be designed as follows. Firstly, we input test images to background model, and a template is selected from the library; secondly, a trimap is obtained from the output of the calculating model; finally, we apply the trimap to initialize Grabcut background model, and the target region can be obtained after the segmentation.

**Figure 2:**
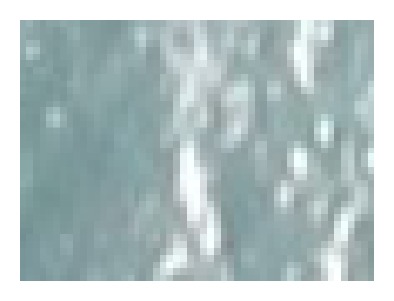
The selected template from the initial template library.

**Figure 3:**
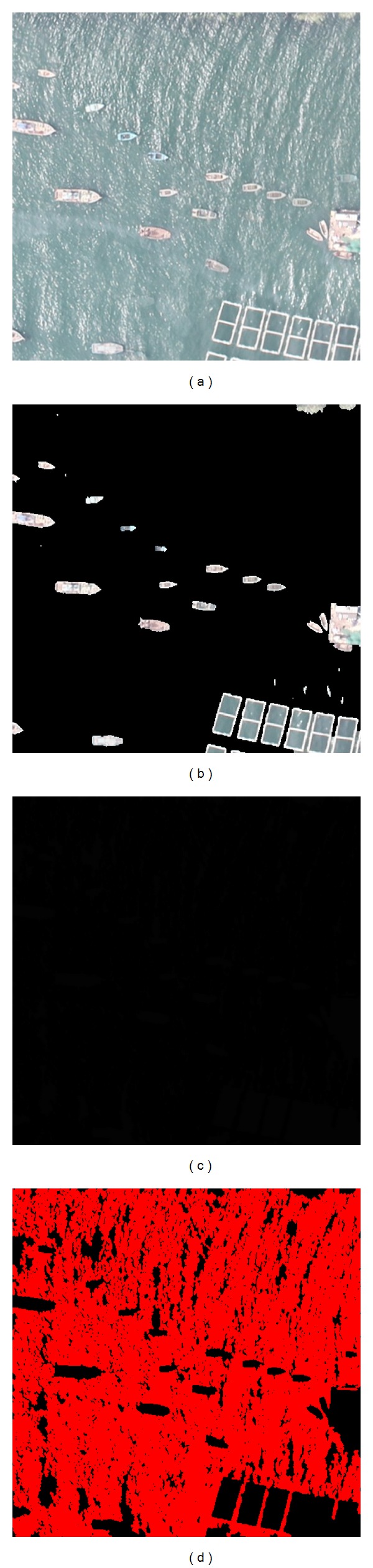
Results of Grabcut based on background model. (a) Original image; (b) result image; (c) output trimap of the background model; (d) red areas of significant show of trimap.

Figure 2 shows the most similar template which is selected from the initial template library. And Figure 3 shows the UAV aerial image and the results of the proposed algorithm on the real world image. With the high resolution of the image, the shape of boats and mariculture zones can be seen clearly. In the result image, 29 candidate regions are detected, wherein there are 18 boat candidates (18 boats), 5 sea debris regions, and 6 mariculture zones. Small bright spots in the image are surface debris which has no effect on the detection of boats. The distance threshold value of template matching algorithm is assigned as 0.07 and only one iteration of Grabcut. The false alarm rate is 17.24%.

**Figure 4:**
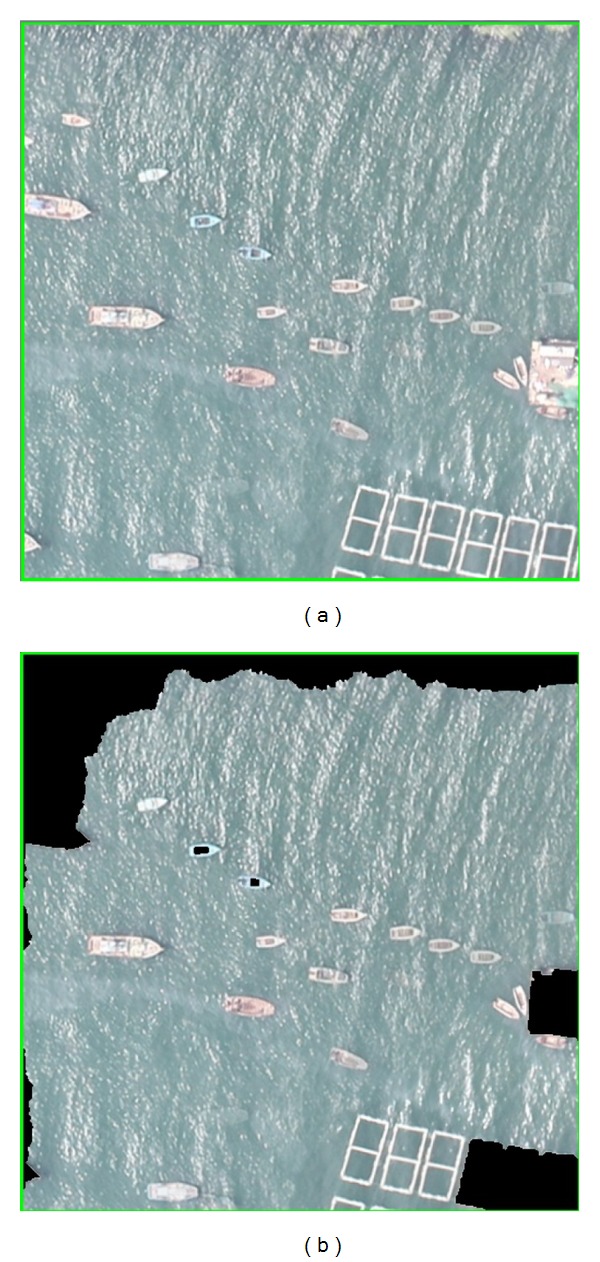
Results of Grabcut algorithm and manual rectangle. (a) Green rectangle is the manual rectangle; (b) result of the 15th iteration.

On the contrast, the original Grabcut is used to perform on the same image, and the manual rectangle and the results are shown in Figure 4. It can be seen that the calculation is convergent at the end of the 7th iteration, and the results of the 7th and the 15th are almost the same. In Figure 4, we cannot calculate the false alarm rate, because there is no ship being segmented from the background by the Grabcut method.

Since the templates in the library have different conditions, we can get better segmentation results for different sea images. Furthermore, one template is not only limited to one kind of image condition. The following experiments are carried on different scenarios. For each scenario, both Grabcut [29] method and our improved method BgCut are applied to the target image, respectively. And the Grabcut representation is the final iteration result.

#### 4.1.1. Scenario: Part of the Ship on the Edge of the Image

**Figure 5:**
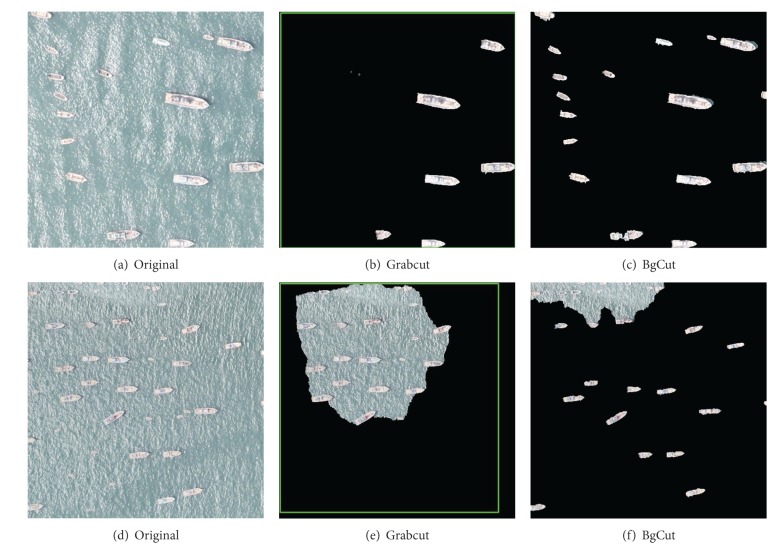
Scenario of Part of the Ship on the Edge of the Image. Results of Grabcut and BgCut.

In practice, the single ship image is segmented from the whole image based on pyramid multiresolving. Consequently, parts of the ships appear on the edge of the image, and the comparison results are shown in Figure 5. It can be seen that the Grabcut algorithm can segment some of ships, while our proposed algorithm can segment more ships and achieve more accurate segmentation results.

#### 4.1.2. Scenario: Ship with Different Sizes

**Figure 6:**
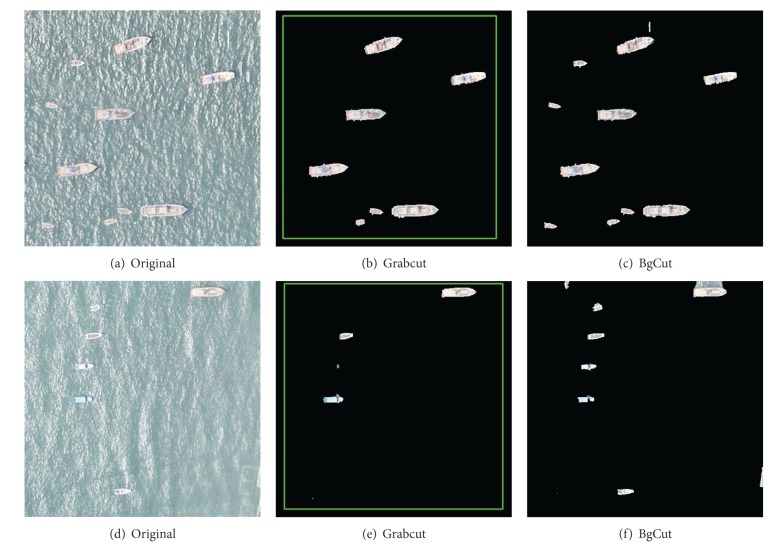
Scenario of Ship with Different Sizes. Results of Grabcut and BgCut.

There are many kinds of ship on the ocean such as vessels and fishing boats. If the ship sizes vary widely, it has a great influence on segmentation. Some smaller ships are often identified as the background, because these ships are similar to the background. The comparison results are shown in Figure 6. The results show that our algorithm can almost identify all the ships under the premise of sacrificing some accurate edge segmentation.

#### 4.1.3. Scenario: Image Blur and Low Resolution

**Figure 7:**
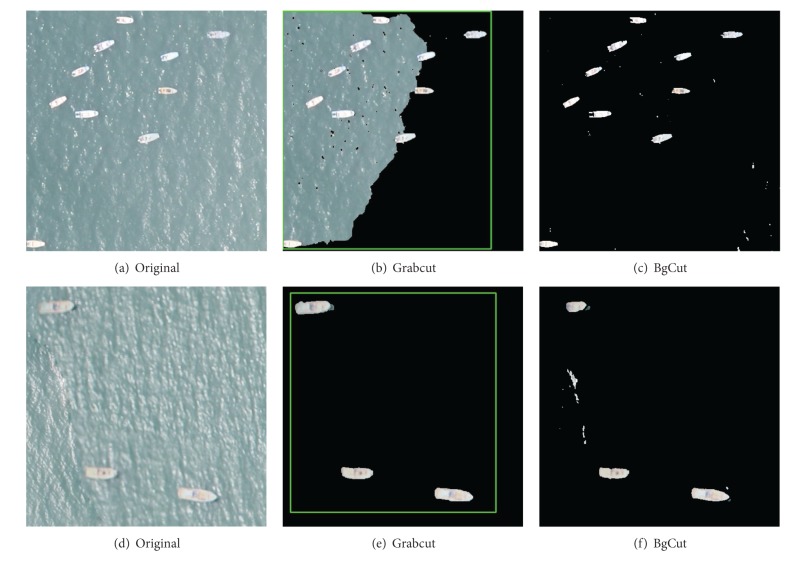
Scenario of Image Blur and Low Resolution. Results of Grabcut and BgCut.

Because of the impact of external distraction factors and jitter of UAV during the data collection, it is easy to make the image blur. Obviously, fuzzy edge has a great influence on unknown pixels classification and identification. The results of Grabcut and BgCut are shown in Figure 7. In this scenario, Grabcut algorithm may have smoother edge segmentation if the ships are not near to the edge of the image. Our BgCut algorithm may have some noise for the sea surface.

#### 4.1.4. Scenario: Reef Edge and Shore Removed Incompletely

**Figure 8:**
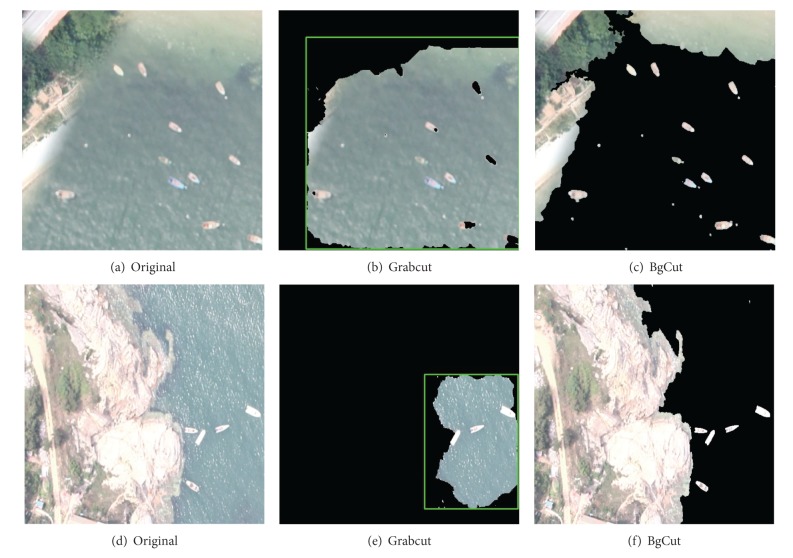
Scenario of Reef Edge and Shore Removed Incompletely. Results of Grabcut and BgCut.

Reef edge or the shore removed incompletely can also appear in the target image. For this situation, the interference also has a great impact on the split. The results of Grabcut and BgCut are shown in Figure 8. Our algorithm has a better effect on processing this kind of image. And the results show that the reef edge and shore will not interfere with the segmentation.

#### 4.1.5. Scenario: Ships Concentration Areas

**Figure 9:**
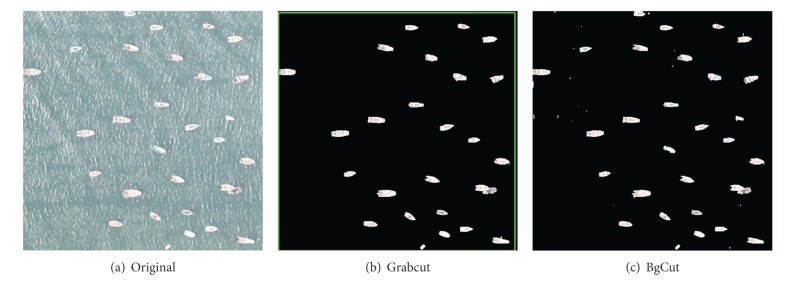
Scenario of Ships Concentration Areas. Results of Grabcut and BgCut.

There are some ships much closer to the mariculture zone than to the reef area. According to the specific conditions, ships tend to gather in designated area. And in the areas of large ships gathered, accurate detection of ship number and the location of the ship are difficult for fishery activities.  Figure 9 shows the results of ship that were identified by Grabcut and BgCut. It is indicated that Grabcut has a good effect on processing ships in gathered area, while our BgCut algorithm has a higher accuracy rate.

#### 4.1.6. BgCut Detection Results of Ships and Mariculture Zone

**Figure 10:**
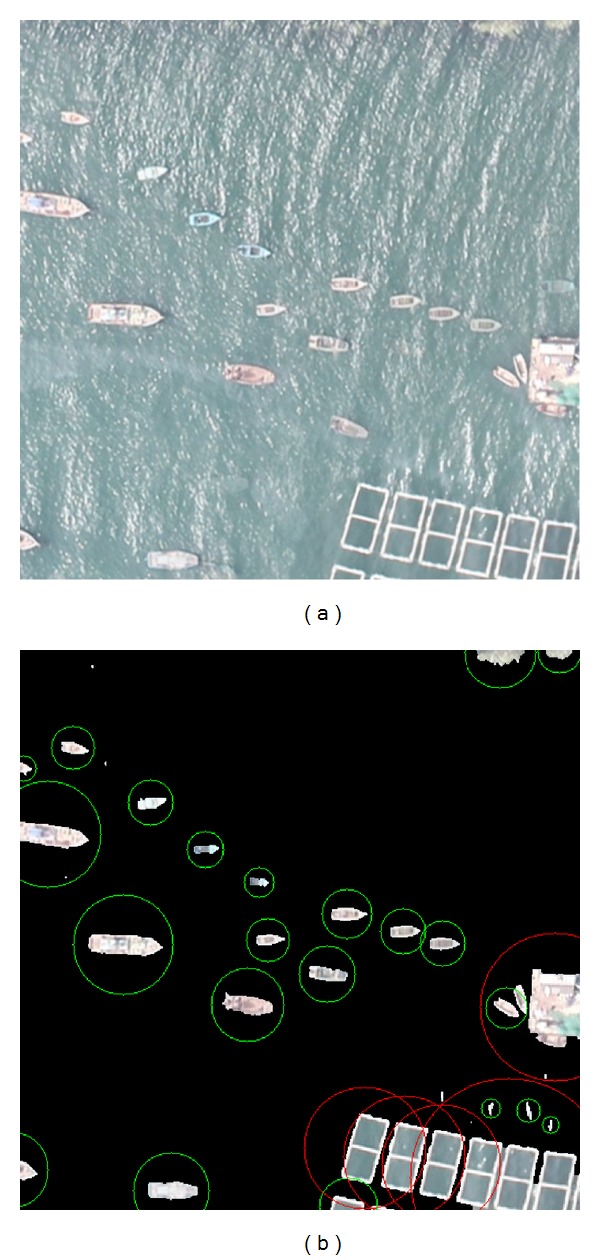
Ships Detection. (a) Original image; (b) detection result: ships in green and mariculture zone in red.

Finally, we will give the BgCut detection results of ships and mariculture zone. The candidate regions are almost full of ships, which provide a good foundation for the realistic ship detection. What we should do is just applying shape texture feature library or some information that can be suitable for detecting ships. As shown in Figure 10 we use a specific threshold to distinguish and filter the shape feature. At last, the ships and mariculture zone are circled by our BgCut algorithm: the fishing boats in green circle and mariculture zone in red circle.

## 5. Conclusion

This paper proposes and designs an effective and efficient approach to automatic ship detection from industrial UAV images. A universal background model based on Grabcut algorithm is introduced to initialize Grabcut and obtain better segmentation results. The proposed algorithm does not need manual interaction during the whole process. It can get accurate ship candidate regions and make the big-data image processing to be an automatic flow. The results of segmentation are precise and the recognition is accurate. Experimental results for aerial images obtained from the East China Sea indicate that out improved BgCut model can perform well and meet the needs of practical industrial conditions. The future work will focus on more in-depth learning to increase accuracy of ship detection and make the parameter of region grow more adaptive.

## Figures and Tables

**Algorithm 1:**
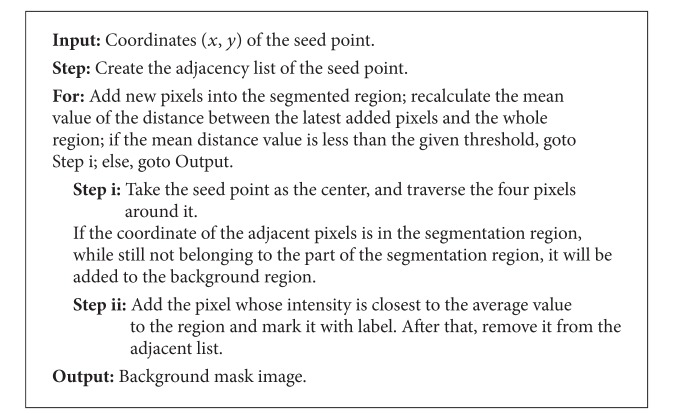
Optimal background region growing flowchart.
